# Identifying the strains of dengue circulating in the western province of Sri Lanka during 2019–2022

**DOI:** 10.1371/journal.pgph.0003150

**Published:** 2024-07-16

**Authors:** Harshi Abeygoonawardena, Kanchana Dassanayake, Jayani Kariyawasam, Teshan Chathuranga, Tharmini Sundralingam, Hansani Gunasekara, Sathyani Wevita, Gayani Premawansa, Sunil Premawansa, Ananda Wijewickrama, Namal Wijesinghe, Varuna Navaratne, Daniela Weiskopf, Alessandro Sette, Chandanamali Punchihewa, Aruna Dharshan De Silva

**Affiliations:** 1 Faculty of Medicine, General Sir John Kotelawala Defence University, Kandawala Estate Ratmalana, Sri Lanka; 2 Genelabs Medical (Pvt) Ltd, Nawala Rd, Nawala, Sri Lanka; 3 Colombo North Teaching Hospital, Ragama, Sri Lanka; 4 Faculty of Science, University of Colombo, Colombo, Sri Lanka; 5 National Institute of Infectious Diseases, Angoda, Sri Lanka; 6 La Jolla Institute for Immunology, Center for Infectious Disease and Vaccine Research, La Jolla, California, United States of America; 7 Department of Medicine, Division of Infectious Diseases and Global Public Health, University of California, San Diego, California, United States of America; Universidad Autonoma de Baja California, MEXICO

## Abstract

A study conducted from July 2019 to May 2022 at several hospitals in the Western Province, Sri Lanka, focused on dengue virus strains during the COVID-19 pandemic. Among 417 febrile patients, 47% were PCR-positive for dengue. Serotyping revealed DENV-1 (12.8%), DENV-2 (46.4%), DENV-3 (37.2%), and DENV-4 (3.6%). Sequencing identified two genotypically distinct variants of DENV-3 and two genotypically distinct variants of DENV-1, while DENV-2 showed a single genotype cluster. Notably, the study found concurrent circulation of two DENV-3 and two DENV-1 genotypes, along with DENV-2, during the pandemic in the area. This data suggests the presence of multiple dengue strains, including several DENV-1 and DENV-3 variants, without major epidemic outbreaks reported in the Western Province. Continuous monitoring and research are essential to understand the dynamics of these dengue strains in the context of the COVID-19 pandemic.

## Introduction

Dengue viruses (DENV) have emerged as the predominant arbovirus globally imposing a substantial public health and economic burden on a worldwide scale [1,2]. Over 2.5 billion individuals reside in regions characterized by a heightened risk of DENV infection, particularly in densely populated urban areas within tropical countries [2,3]. The spectrum of symptoms associated with DENV infection varies, encompassing a febrile illness resembling flu-like symptoms, with or without warning signs, to severe manifestations such as plasma leakage, bleeding tendencies, or organ dysfunction [4]. Despite the availability of a licensed vaccine against DENV in various countries, it is yet to be adapted by national vaccination programs. Furthermore, the absence of a specific antiviral treatment for DENV infection poses a significant challenge in managing and mitigating the impact of the disease [2,5,6].

Dengue viruses (DENV) are part of the Flavivirus genus within the Flaviviridae family. This viral group comprises of four distinct serotypes, namely DENV-1 through DENV-4, which exhibit both immunological and genetic uniqueness. Further classification reveals that each DENV serotype encompasses a minimum 3 different genetically divergent genotypes. Although all four serotypes are prevalent in regions where DENV is endemic globally, the genotypes within each serotype often display geographical segregation.[2,7]. However, within each DENV serotype, distinct genotypes emerge, and these genotypes typically display geographic separation. This geographic divergence in genotypes within each serotype adds an additional layer of complexity to the understanding of DENV distribution and epidemiology. In Sri Lanka, all four DENV serotypes are endemic and 186,101 cases were reported in 2017 according to the data available in the Epidemiology unit Sri Lanka [8]. The serologically confirmed first case of DF was reported in 1962 in Sri Lanka [9]. First major outbreak of Dengue has been reported in 1989 associated with dengue 3 serotypes [9]. Dengue continues to cause regular epidemics and still remains a public health problem for Sri Lankan authorities. Prior to 1989, the dengue epidemiological landscape in Sri Lanka was marked by the regular occurrence of all four dengue serotypes, with a relatively low incidence of dengue hemorrhagic fever (DHF). However, following the year 1989, there was a noteworthy and significant surge in the number of reported cases of DHF [7]. This shift in the epidemiological pattern highlighted a substantial change in the severity and clinical manifestation of dengue in Sri Lanka during that period. The study in 2002 by Messer [10] concluded that the surge in dengue hemorrhagic fever (DHF) cases in Sri Lanka was not adequately explained by an increase in virus transmission or a shift in circulating dengue serotypes. Despite the presence of all four serotypes and sustained transmission, these factors alone were insufficient to account for the epidemic emergence of DHF during that period [10]. The study suggested that additional factors or mechanisms might have played a crucial role in the escalation of DHF cases in Sri Lanka. [10,11]. The historical data indicates that the population in Sri Lanka had experienced exposure to dengue infections in the past without witnessing outbreaks of dengue hemorrhagic fever (DHF), which is a severe manifestation of dengue. However, a significant turning point occurred in 1989 when Colombo, the capital of Sri Lanka, faced its initial large outbreak of DHF. This outbreak marked a substantial departure from previous patterns, highlighting the emergence of a more severe form of dengue in the region.

Subsequent to the 1989 outbreak, the reported incidence of dengue in Sri Lanka has demonstrated a consistent upward trend, with increments occurring approximately every 3 to 5 years. This escalation has been accompanied by a gradual geographical spread, encompassing various regions across the country. Notably, until the year 2009, the annual reported cases remained below 15,000. However, a significant surge was observed in 2009, surpassing 40,000 cases, and this increase was attributed to the emergence of Dengue Virus (DENV) serotype 1. The changing dynamics underscore the evolving nature of dengue epidemiology in Sri Lanka. [12] [13]. Between 2009 and mid-2016, Dengue Virus serotypes 2 (DENV-2) and 3 (DENV-3) were conspicuously absent in the detected cases, despite these serotypes being the predominant ones circulating before 2009 in Sri Lanka [10]. The absence of DENV-2 and DENV-3 during this period marked a notable shift in the prevalent serotypes compared to the pre-2009 era. [11]. The most significant dengue epidemic on record occurred in 2017, witnessing a staggering 186,101 reported cases and 440 fatalities. Intriguingly, this surge in dengue cases coincided with the reappearance of Dengue Virus serotype 2 (DENV-2), highlighting a correlation between the resurgence of this particular serotype and the heightened epidemic impact in that year [14]. Despite a decrease in reported cases in 2018 (51,659 cases) compared to the previous year, there was a renewed upswing in 2019, witnessing 105,049 reported cases. The dominant serotype were dengue virus serotype 2 (DENV-2) in 2019. This resurgence in 2019 reflected a notable increase compared to the preceding year, underscoring the dynamic nature of dengue incidence in Sri Lanka during that period. [11].

In this research endeavor, our focus extended to the continuation of laboratory-based surveillance, aiming to furnish crucial insights into the molecular epidemiology of Dengue Virus (DENV) strains present in the Western Province of Sri Lanka from 2019 to 2022.

## Materials and methods

### Ethics statement

Ethics approval for this study was obtained from Ethics Review Committee (ERC), Faculty of Medicine, General Sir John Kotelawala Defence University (Reference: RP/2018/02) and Ethics Review Committee, Faculty of Medicine, University of Colombo (Reference: EC 15–095). Samples collected from 1st July 2019 to 31st May 2022 was approved by the EC 15–095 approval from 23rd July 2015 and the approval was extended annually up to 16th June 2023.

Initial Ethical approval for RP/2018/02 was taken 26th April 2018 and extended annually until 30th March 2022. All the ERC approvals and extensions are available upon request.

### Study area and sample collection

Four hundred and seventeen consenting patients presenting with febrile illness (suspected dengue) were recruited from the Colombo North Teaching Hospital (CNTH), National Institute for Infectious Diseases (NIID) and University Hospital-Kotelawala Defence University (UHKDU) of the Western Province from 1^st^ July 2019 to 31^st^ May 2022, whom were enrolled in the study following informed written consent. The initial blood sample was obtained between day 1 and 5 of illness (day 1 was considered as first day of fever) from all the patients. In diagnosing patients, the 2009 World Health Organization (WHO) dengue classification scheme and case definition were employed. Comprehensive data, encompassing demographic attributes such as age, gender, and race, as well as clinical manifestations including the day of fever onset, bleeding occurrences, plasma leakage, abdominal pain, and hypotension, were gathered. Additionally, routine hematological and biochemical laboratory test results, such as full blood count and liver function tests, were compiled as part of the information collection process. All samples were tested by Reverse transcriptase polymerase chain reactions (RT-PCR) and/or Real time-PCR tests to determine dengue positivity and serotype. Sequencing was carried out on samples with adequate viral load followed by phylogenetic analysis using the Geneious software. Sequencing data were analysed by Ion Torrent Suite.

### Serological detection

In this study, dengue was diagnosed by NS1 antigen testing on admission (one step SD Bioline dengue NS 1 antigen test, Alere SD, USA, 88.65% sensitivity and 98.75% specificity up to day 9 of fever) and acute-phase blood samples were collected in EDTA-containing tubes between days 1 and 5 of illness. Plasma was prepared within 6 h of collection and stored at −80°C until use.

### RNA extraction and reverse-transcription polymerase chain reaction (RT-PCR)

RNA was extracted from all serum samples using QIAamp Viral RNA Mini Kit (Qiagen, Germany) according to the manufacturer’s protocol. Target viral RNA was converted to a DNA copy (cDNA) and conventional PCR (RT-PCR) reaction with samples separated on a gel was routinely performed according to the protocol described in the paper [15]. The conventional PCR was carried out for all collected 417 samples and the samples that were negative were further analyzed with real-time PCR by using a commercially available VIASURE Real-time PCR (CerTest Biotec, S.L., Zaragoza, Spain). The test was carried out in one step real time RT format where the reverse transcription and the subsequent amplification of specific target sequence occur in the same reaction well. The isolated RNA target was transcribed generating complementary DNA by reverse transcriptase which was followed by the amplification of 3’Non coding region (DENV), using specific primers and fluorescent–labelled probe. The kit is based on 5’ exonuclease activity of DNA polymerase. Dengue virus RNA targets are amplified and detected in FAM channel and the internal control HEX channel in Rotor-gene instrument.

### Sequencing and phylogenetic analysis

**Fig 1:**
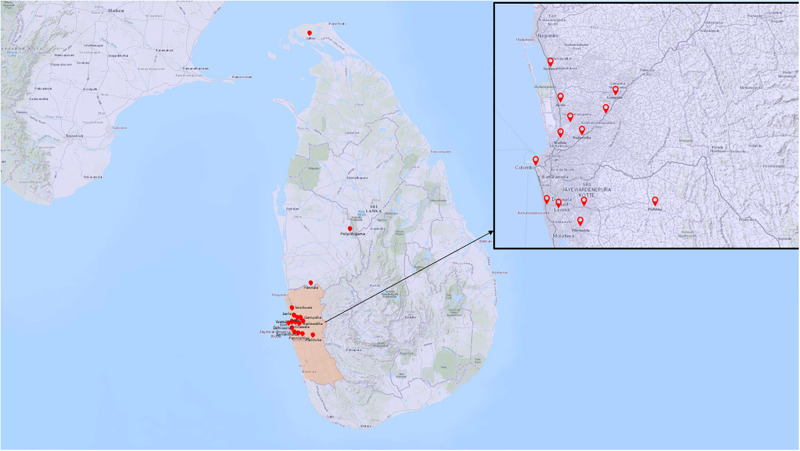
The location of sequenced isolates. Base map source: https://www.usgs.gov/maps/international-geomagnetic-reference-field-2005.

Sequencing was carried out on samples with adequate virus titer followed by phylogenetic analysis. Accordingly, nine isolates from DENV-3, nine isolates from DENV-1, and seven isolates from DENV-2 were sequenced using NGS and analysed by using Torrent suite. The complete genome of DENV1: NC_001477, DENV2: NC_001474, DENV3:NC_001475) isolates were initially searched for similarity/blast using Geneious Prime software Version 2021.2.2. DENV-1,2,3 sequences with high similarity were selected for further analysis. Specifically, sequences were aligned using ClustalW and nucleotide sequences identity matrix. The amino acids substitutions were also analyzed using the same method. Phylogenetic analysis and distance calculations were performed using the Geneious Prime software Version 2021.2.2 with the Neighbor-Joining method of the Maximum Composite Likelihood model, gamma-distributed rates among sites with 1,000 bootstrap replicates. The location of the sequences are shown in the Fig 1. Amino acid substitution among the newly isolated viruses were also analyzed in their protein expression levels compared to reference strains (DENV1: NC_001477, DENV2: NC_001474, DENV3:NC_001475). Genotypes are defined based on the nucleotide divergence of 6%–8% within each serotype [16]. Within a genotype, the group of DENV strains which form phylogenetically distinct monophyletic clusters with bootstrap support of at least 75% [16].

## Results

### Serotyping

All samples (417) were tested by conventional PCR (RT-PCR) with samples separated on a gel. Only 170 samples dengue suspected samples were positive. Then all negative samples (417–170 = 247) were tested by Real time PCR, among them 26 were positive for real time PCR, for a total of 196 positives (196/417). The serotyping showed 12.8% (25/196), 46.4% (91/196), 37.2% (73/196) and 3.6% (7/196) of the positives were DENV-1, DENV-2, DENV-3 and DENV-4 respectively. None of the samples became positive for more than one serotype in our testing. Among 196 samples, 85 dengue positive plasma samples were sent for sequencing, then due to low viral load and inadequate plasma, only 25 samples could be sequenced. Of the 25/196 DENV1, only 09/25 DENV 1 samples were able to be sequenced. Of the 91/196 DENV2, only 07/91 DENV2 samples were able to be sequenced. Of the 73/196 DENV3, only 09/73 DENV3 samples were able to be sequenced. Of the 7/196 DENV4, none of the samples were able to be sequenced.

### Phylogenetic analysis

Patient samples of DENV were sent for DENV NGS sequencing. Sequences were aligned using Geneiuos software and Phylogenetic tree was developed using Geneious and tree builder was constructed using Neighbor joining method. The genome coverage of isolates were 80%-100%, phylogenetic analysis was done using the available genome sequence (80–100%).

**Fig 2:**
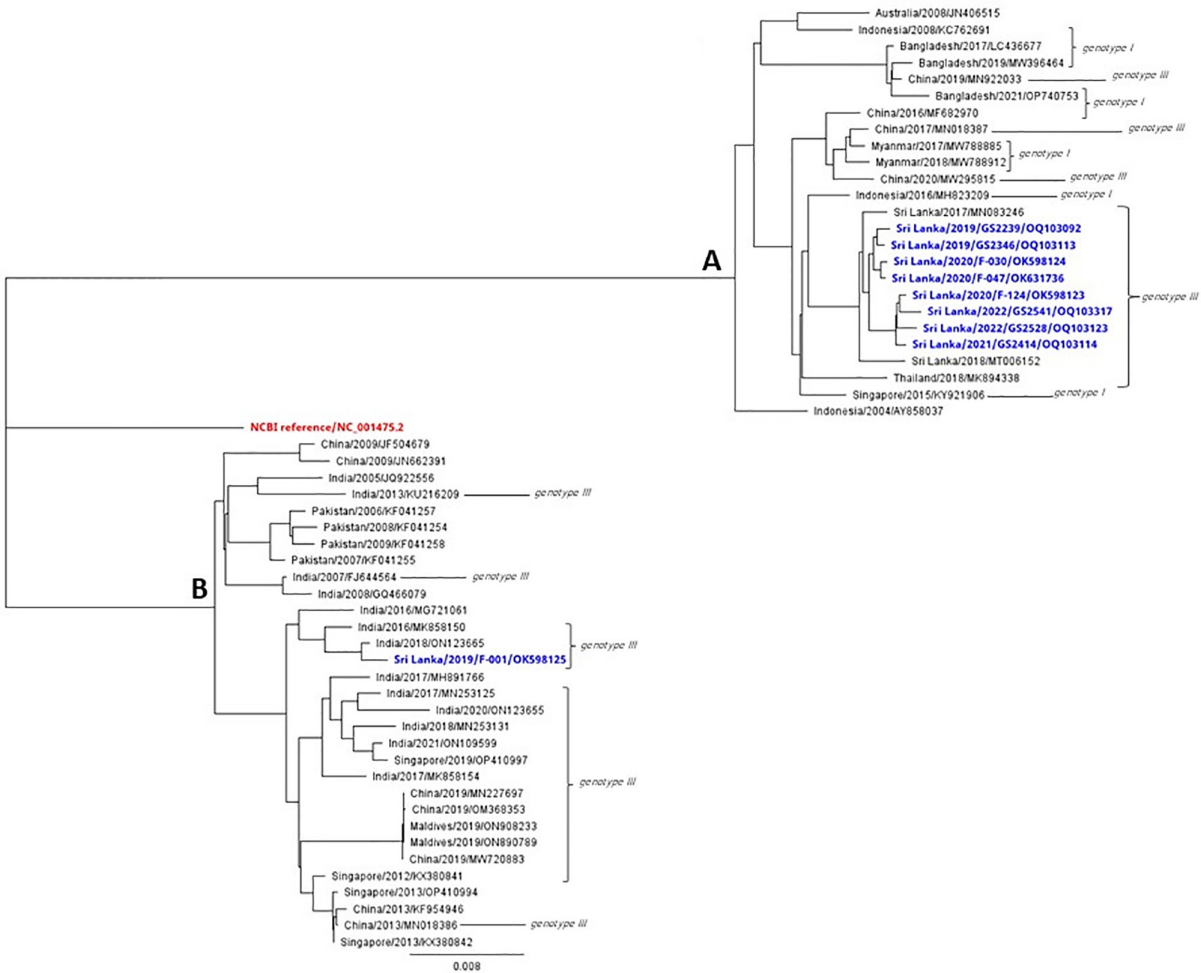
DENV3 virus strains phylogenetic tree.

At this stage depending on the virus titer we could sequence only nine samples of DENV 3. These nine samples were located under two branches of the phylogenetic tree (Fig 2). We did a detailed analysis based on the identity percentage under the two branches. Eight samples were under the same branch while one is different. Based on these findings, we could speculate that these eight samples of the same phylogenetic branch (Fig 2), are from the previous DENV 3 strains that circulated in 2017 and 2018. It suggests that the same strain is circulating from 2019, 2020, 2021 and 2022. It probably came to Sri Lanka from Singapore and Indonesia according to our sequence analysis. There are no new dengue sequences, other than the ones published in this manuscript, from Sri Lanka after 2018.

**Fig 3:**
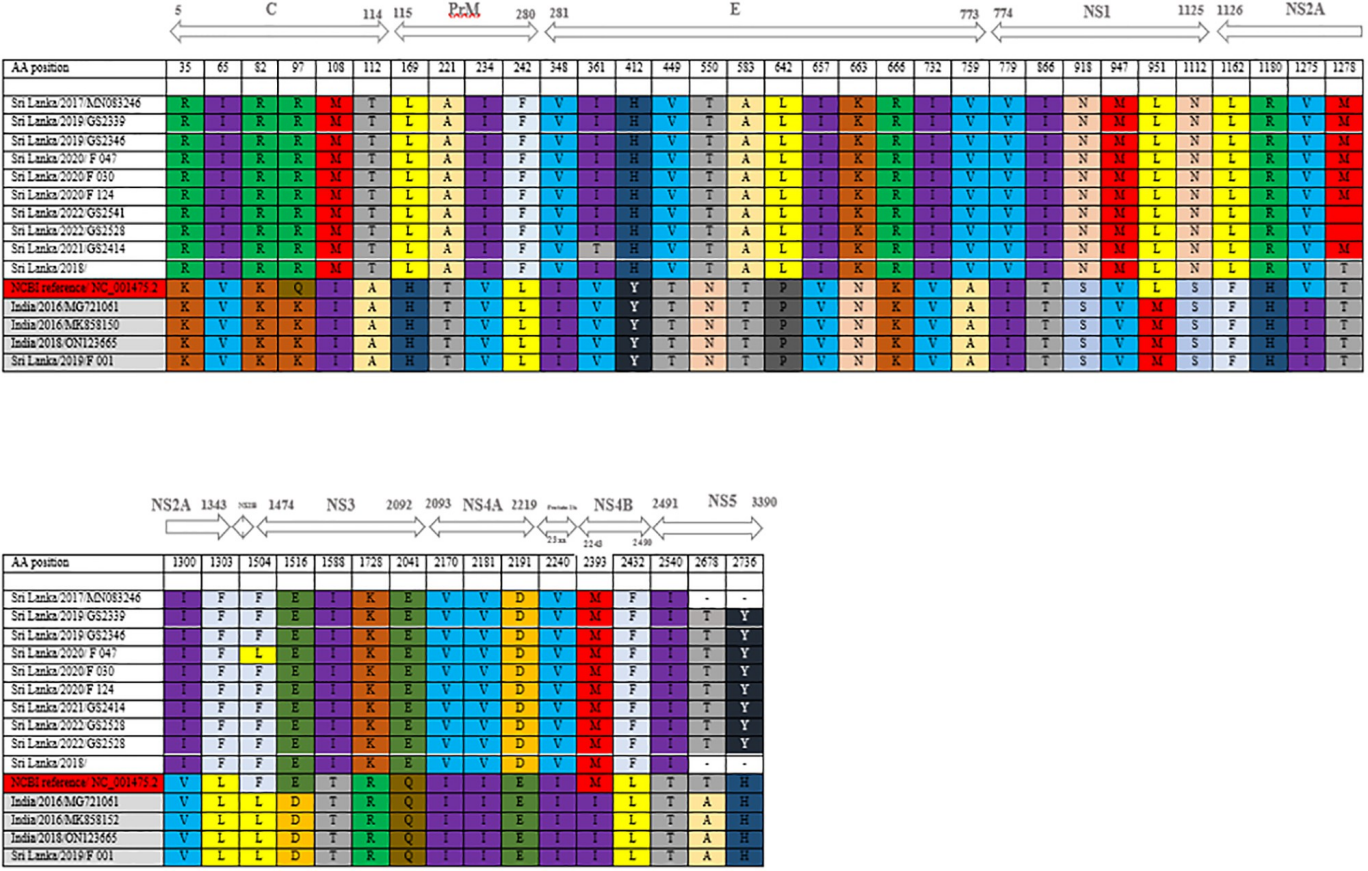
Amino acid substitutions of DENV3.

When studied in detail, other DENV3 viruses show close resemblance to strains isolated in India during 2016 and 2018 (Fig 2). This data shows different genotypes from each of the different serotypes circulating during the same period and this has not been observed in Sri Lanka since 2008. There were only one or two dominant genotypes from a serotype from 2009 onwards. Furthermore, the F-001(OK598125) genotype has a distant relationship with the strains reported in 1989/1997 with a 96% similarity in nucleotide bases. This suggests that this strain may have been circulating in the Indian subcontinent for some time. According to the analysis of amino acids substitution, two clusters were noted (Fig 3).

**Fig 4:**
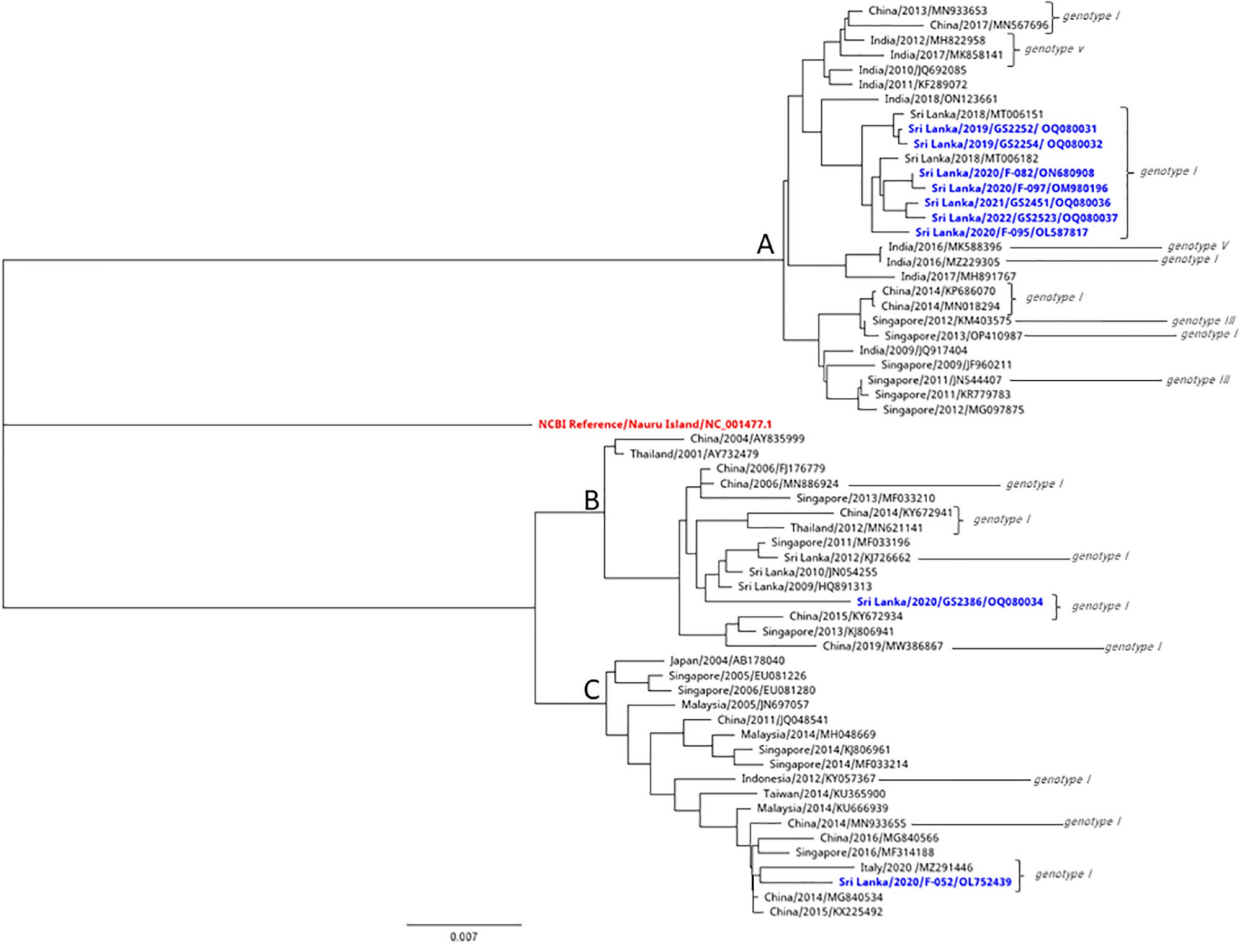
Phylogenetic analysis of DENV1 strains.

**Fig 5:**
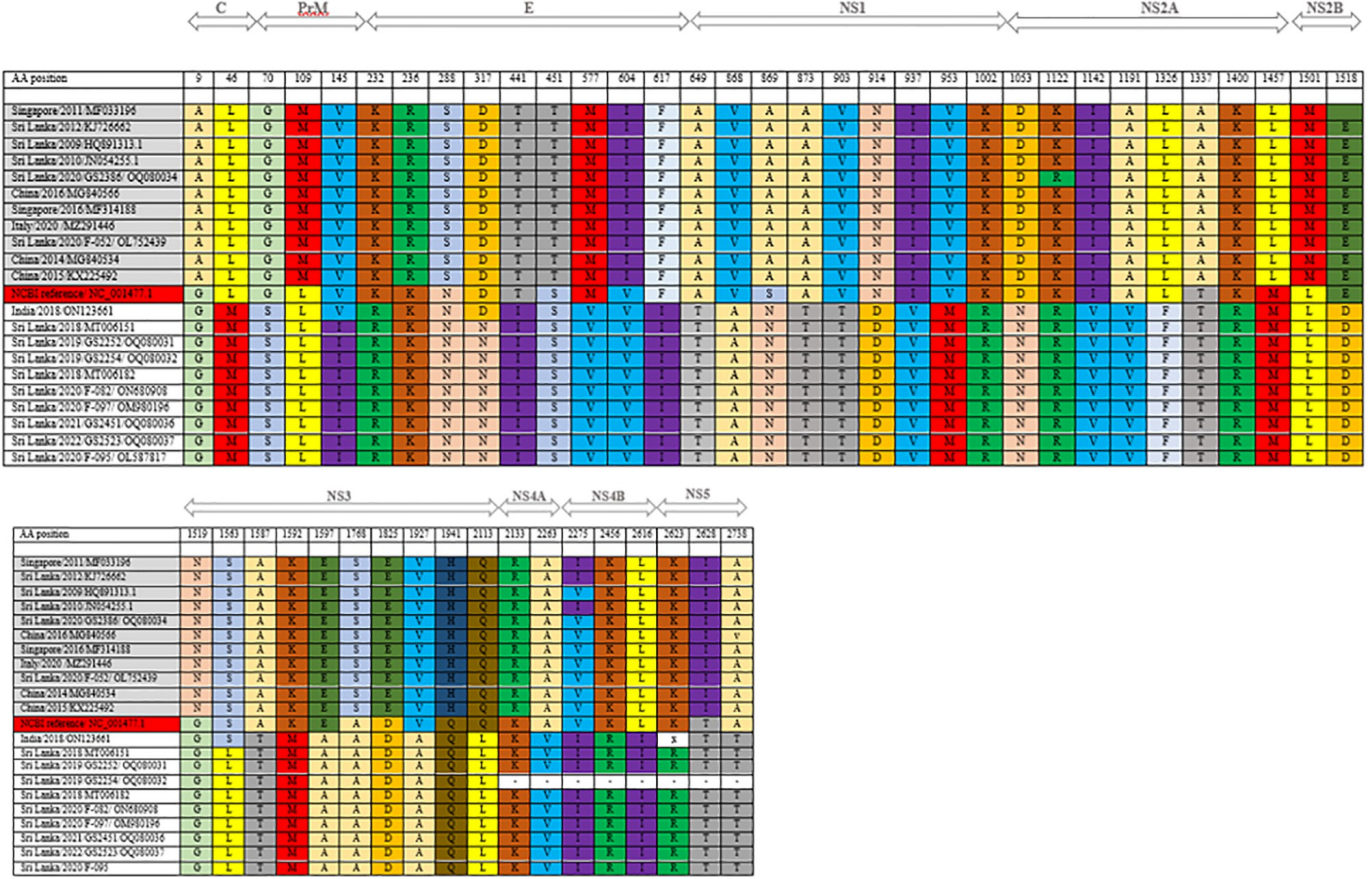
Amino acid substitutions of DENV 1.

**Fig 6:**
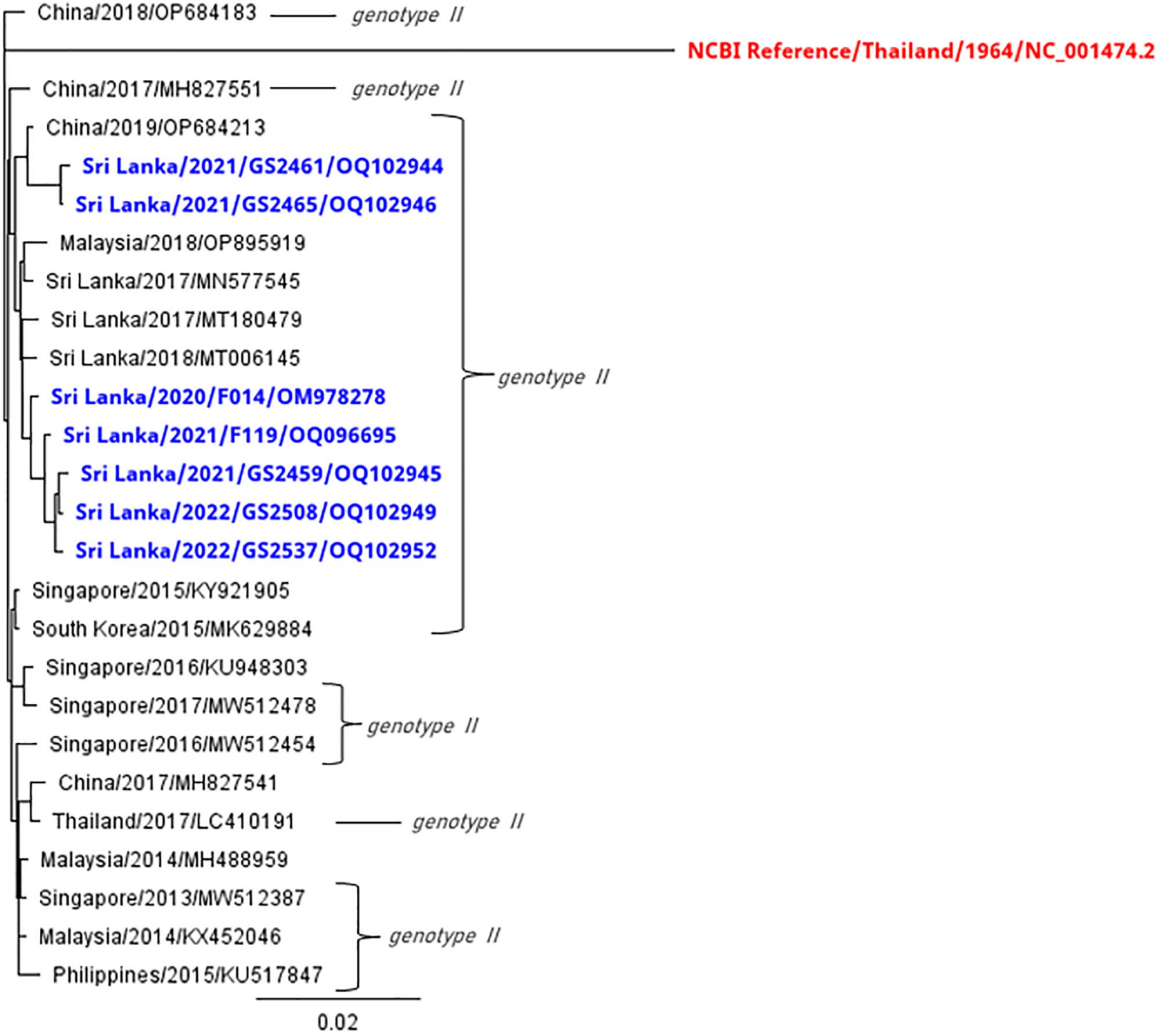
Phylogenetic analysis of DENV2 strains.

Due to low virus titers we could only sequence nine samples of DENV 1 (Fig 4). Seven samples of DENV 1 are similar to genotype that circulated in Sri Lanka in 2018. One genotype is similar to a genotype isolated in China 2014/2015 and the other one is similar to 2009/2010 reported genotypes in Sri Lanka. Therefore, DENV3 and DENV1 have different genotypes from the same serotype circulating during the same period. Three types of DENV1 genotypes are currently circulating in the country. One of DENV 1 genotype which circulated in 2018 still exists and continues to circulate, while the other one has a distant relationship with the genotype reported in 2009/2010 which is 97% similar in their nucleotide sequences. This suggest that previously reported genotype is genetically changing and F-052 (OL752439) has 99% similarity to reported cases in China (2014/2015). According to the amino acid substitution (Fig 5), it shows that two clusters of amino acids substitution which can be seen as the same pattern in the nucleotide bases like DENV-3. Among the two clusters, there is one genotype which differs from these two clusters of amino acids showing a different amino acid pattern to the other genotypes. Currently circulating DENV2 is similar to the strains that have been circulating from 2017 onwards (Fig 6).

In summary, two genotypically distinct variants were identified from sequencing of the DENV-3 and three genotypically distinct variants were identified in DENV-1 containing samples. DENV-2 strains were similar to the genotypes reported in Sri Lanka previously. DENV-4 samples did not have an adequate viral load for sequencing. One of the identified DENV-3 variants was similar to that reported in 2017/2018 in Sri Lanka while the other variant was similar to the variant reported in India in 2016/2018. One of the identified DENV-1 variants was similar to the 2018 genotype found in Sri Lanka while the other was similar to the variant reported in China in 2014/2015. Sequencing was carried out on samples from UHKDU and CNTH. From the second quarter of 2020, NIID did not have dengue patients because it was a converted to a COVID-19 treatment hospital and there were no dengue patients until 2021. Other samples we had collected prior to 2020 were stored in the lab, but due to limited storage and freezer problems the virus samples may have deteriorated and the viral load would be inadequate for sequencing.

No new variant was reported for the DENV-2 tree, suggesting that the old DENV-2 which caused the epidemic in 2017 is still circulating. The accession numbers of all the sequences are given in the Table 1.

**Table 1:** Serotype details along with GenBank accession numbers of dengue viruses from isolated sequences.

Srl. No.	Year/month	Lab ID no	Serotype detected	GenBank Accession number	Location/area
**1**	2020/09	F-095	DENV-1	OL587817	Pannipitiya
**2**	2020/06	F-052	DENV-1	OL752439	Piliyandala
**3**	2020/09	F-097	DENV-1	OM980196	Piliyandala
**4**	2019/07	GS-2252	DENV-1	OQ080031	Wattala
**5**	2019/07	GS-2254	DENV-1	OQ080032	Polpithigama*
**6**	2020/08	F-082	DENV-1	ON680908	Piliyandala
**7**	2020/08	GS-2386	DENV-1	OQ080034	Kadawatha
**8**	2021/10	GS-2451	DENV-1	OQ080036	Gampaha
**9**	2022/03	GS-2523	DENV-1	OQ080037	Kadawatha
**10**	2021/11	GS-2459	DENV-2	OQ102945	Seeduwa
**11**	2021/11	GS-2461	DENV-2	OQ102944	Jaela
**12**	2020/02	F-014	DENV-2	OM978278	Dehiwela
**13**	2021/12	GS-2465	DENV-2	OQ102946	Wattala
**14**	2021/01	F-119	DENV-2	OQ096695	Jaffna*
**15**	2022/02	GS-2508	DENV-2	OQ102949	Ragama
**16**	2022/05	GS-2537	DENV-2	OQ102952	Ragama
**17**	2019/12	F-001	DENV-3	OK598125	Colombo Fort
**18**	2020/06	F-030	DENV-3	OK598124	Pannala*
**19**	2020/06	F-047	DENV-3	OK631736	Bellantota
**20**	2020/12	F-124	DENV-3	OK598123	Padduka
**21**	2020/01	GS-2346	DENV-3	OQ103113	Wattala
**22**	2021/03	GS-2414	DENV-3	OQ103114	Kadawatha
**23**	2019/07	GS-2238	DENV-3	OQ103092	Kadawatha
**24**	2022/03	GS-2528	DENV-3	OQ103123	Kirillawala
**25**	2022/05	GS-2541	DENV-3	OQ103317	Ragama

* Town is not in the Western Province, but the patient frequently travelled to Western Province and admitted to a hospital in the study area.

### Comparison of base pair similarity among sequences

Similarities between all samples of DENV3 and previously published Sri Lankan strains are shown in a matrix (S1 Table). Strains F-30 (OK598124) and GS-2346 (OQ103113) showed the highest similarity of 99.892%. F-001 (OK598125) showed the lowest similarity between itself and the other sequences of around 91%.

In DENV1 sequences, amino acid sequences similarities between isolates and previously published Sri Lankan strains are shown in a matrix (S2 Table). F-0052(OL752439), 2009 and 2010 reported viruses showed 90%-91% similarity. Fig 4 clearly shows the F-052 (OL752439), GS2386 (OQ080034), KJ 72662 sequence from Sri Lanka in 2012 and HQ891313 sequence from Sri Lanka in 2009 showing equal similarity. There are no noted separate clusters in the DENV2 strains despite having a number of amino acid substitutions (Fig 5).

## Discussion

**Fig 7:**
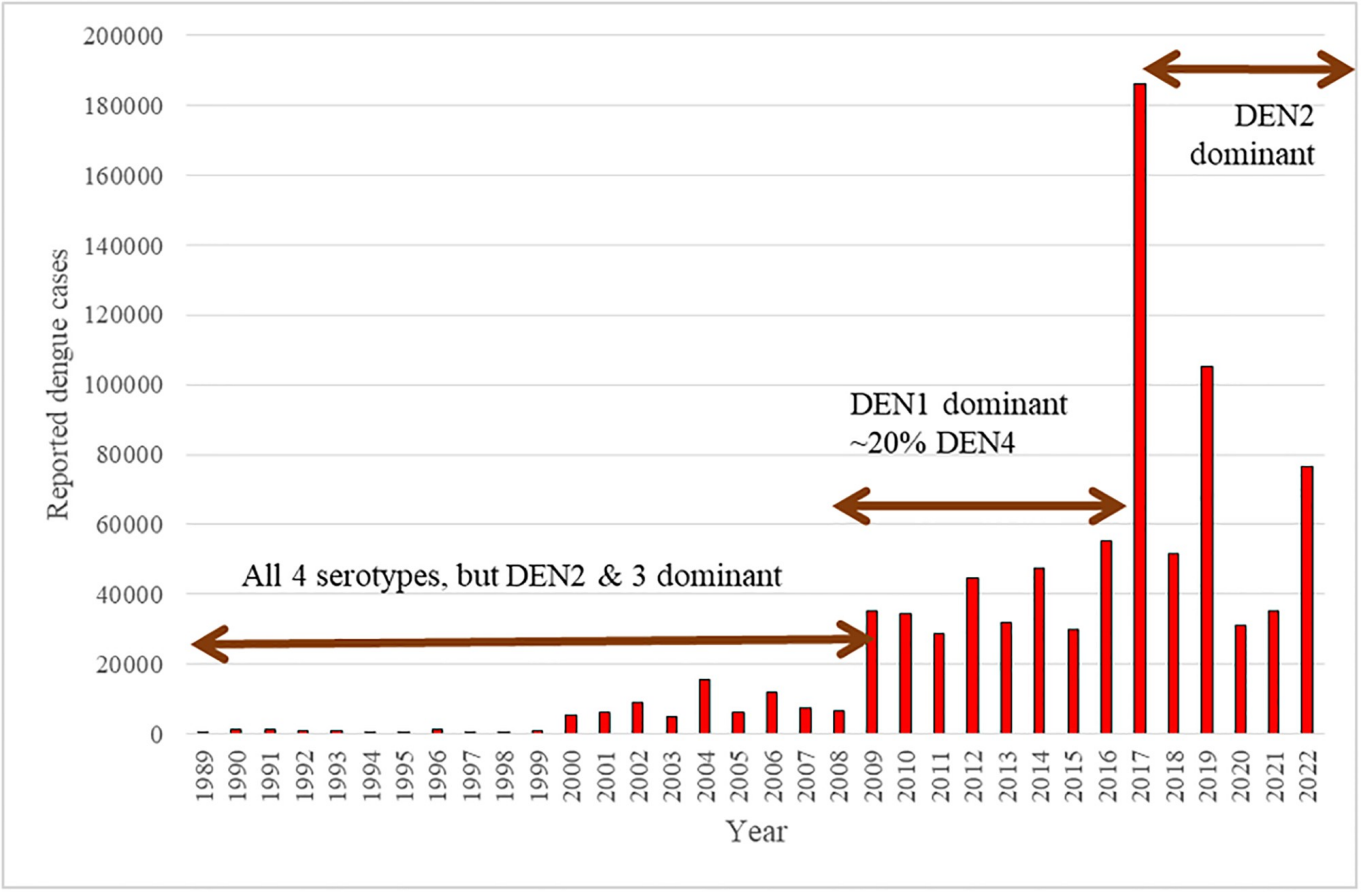
Reported dengue cases from 1989 to 2022 with dominant dengue serotype. Data obtained from the National Dengue Control Unit, Ministry of Health, Sri Lanka.

Our study provides a background investigation of the circulating serotypes of dengue viruses (DENV) in the Western Province of Sri Lanka from July 2019 to May 2022, coinciding with the COVID-19 pandemic. During this period, all four serotypes of DENV (DENV-1, DENV-2, DENV-3, and DENV-4) were identified in the region. DENV-2 and DENV-3 were observed as the most common serotypes in this area with DENV-1 being less common (12%) and DENV-4 being found in very small numbers (3.6%). Interestingly, despite numerous dengue outbreaks reported in Sri Lanka historically (Fig 7), there was no outbreak during the COVID-19 pandemic. The last significant dengue outbreak occurred in 2017, during which there was a considerable increase in dengue cases and related deaths (reported 186,101 cases with 440 deaths), putting tremendous pressure on the country’s healthcare resources. The incidence of dengue cases surged across the entire country, with Nuwaraeliya district having the lowest incidence rate experiencing a threefold increase compared to the average of the previous five years [14]. The outbreak predominantly impacted older schoolchildren and young adults involved in the workforce. There were notable differences in the age-specific incidence rates observed among various provinces in Sri Lanka in the year 2017 [14].

The reasons behind the outbreak in 2017 have been studied, the entomological and climatic factors, excluding rainfall, failed to account for the heightened incidence. Despite the usual association of rainfall with mosquito breeding and its potential role in dengue outbreaks, it did not fully explain the increased cases. Notably, the district most impacted, Colombo, exhibited a prevalence of the DENV2 strain of the dengue virus. This suggests that the specific viral strain circulating in the region might have contributed to the severity and extent of the outbreak[14]. Throughout the history, the incidence of dengue has been marked by seasonal outbreaks that disproportionately impact urban areas, with the Colombo district being particularly conspicuous in this regard. The Colombo district, comprising 11% of the country’s population [17] stands out due to its highest population density. Additionally, with an average of one million people commuting in and out of the city daily, Colombo serves as the commercial and administrative hub of the country. These factors make the district more vulnerable to dengue outbreaks. [18]. Entomologic surveys have shed light on the breeding patterns of the primary dengue vector, *Aedes aegypti*, in Sri Lanka’s Western Province and more than half of the vector breeding sites have been found to be due to discarded items such as containers and tires [14]. This finding highlights the influence of poor container waste management practices in providing conducive breeding grounds for dengue-carrying mosquitoes. Inadequate disposal of such items allow rainwater to collect, creating ideal conditions for *Aedes aegypti* to breed and multiply, exacerbating the risk of dengue transmission. The impact of dengue extends beyond Sri Lanka’s borders, as the global prevalence of the disease is significantly affected by the vector-to-host ratio. This ratio, referring to the abundance of mosquito vectors relative to the susceptible human population and plays a pivotal role in dengue epidemiology worldwide [19–21].

The main question remains as to why there was no outbreak, during COVID-19 pandemic despite the circulating multiple strains. Dengue fever requires a multifaceted approach that considers the interplay between demographic factors, urbanization and travel, plus waste management practices. Implementing effective vector control strategies and promoting responsible waste disposal programs are critical steps in mitigating the burden of dengue, both within Sri Lanka and on a global scale. During the pandemic in Sri Lanka, there were multiple stretches of lockdowns and people were unable to travel with schools closed plus working from home being allowed where possible. This limitation in travel gave more time for people to clean their gardens and houses ensuring better waste management. In addition, international travel was almost at a standstill severely limiting any new dengue strains from entering the country. These factors undoubtedly affected the dengue transmission in Sri Lanka during that time and maybe the reason for not having an outbreak at that time. Dengue cases in Colombo experienced a significant decline of 79.4% from 2019 to 2020. [22]. The implementation of lockdown measures, particularly the closure of schools, appeared to exert a notable influence on the reduction of dengue cases. In contrast, the impact of vector indices was limited. The circulating DENV serotypes exhibited no change, with DENV2 maintaining its prevalence at 65% by early 2022. This pattern closely resembled the frequencies observed at the end of 2019 [22], further shown by our data.

In numerous countries, the occurrence of serotype switches in circulating dengue viruses has been linked to severe epidemics, particularly when the population is exposed to a new serotype. It is important to note that host immunity developed against one dengue serotype provides only partial protection against other serotypes [12,23,24]. In fact, the presence of heterotypic antibodies may even exacerbate the inflammatory response when a person is infected with a different serotype. This phenomenon underscores the complexity of dengue infections and the potential for severe outcomes when new serotypes are introduced [25] and the international travel limitations reduced the chances of this happening.

In Sri Lanka, island wide dengue virological surveillance is been conducted mainly through various research studies but in a haphazard manner. This needs to be carried out in a more consistant and organized manner. A study focusing on a large epidemic in 2009 [12] revealed that DENV-1 was the dominant circulating serotype. This trend persisted in subsequent epidemics in 2010 and 2012 [26]. During the period between 2003 and 2006, [27] it was reported that dengue transmission was primarily attributed to serotypes DENV-2 and DENV-3. After 2008, the number of DENV-1 cases progressively increased [12], supplanting the transmission of DENV-2 & DENV-3 infections. This shift in dominance might have contributed to a decline in population immunity against DENV-2 & DENV-3. While limited virologic surveillance data from the Colombo district indicated a prevalence of DENV-2 in 2017 samples, it is essential to exercise caution when generalizing this finding to the entire country [14]. The dynamics of dengue serotypes can vary across different regions, and comprehensive national virologic surveillance is crucial for obtaining a more accurate and representative picture of the circulating serotypes in Sri Lanka.

According to our most recent results, during 2020 the serotypes DENV-1, DENV-2, DENV-3 and DENV-4 are all found in circulation. In 2009, a sudden surge in dengue infections in Sri Lanka occurred concurrently with the emergence of a new genotype of DENV-1[12]. The 2012–2013 dengue epidemic in Sri Lanka was driven predominantly by DENV-1 with approximately 15% of dengue cases caused by DENV-4 [18]. After the extensive outbreak in 2017, the prevailing DENV serotype continued to be DENV2 until late 2019 [22]. In 2019, a total of 114,240 cases were documented, and the number of cases exhibited a gradual rise towards the latter part of the year [22]. Notably, DENV3 started to emerge towards the end of 2019, coinciding with the upswing in the number of cases. By the end of December 2019, DENV3 accounted for 28.9% of infections in the Colombo district [22]. Our study revealed a prevalence of DENV-2 and DENV-3 serotypes, which are currently dominant and actively circulating in the region. In contrast, DENV-1 and DENV-4 strains were found to be circulating in only a limited number of cases.

In this study, we have sequenced 09 isolates of DENV-3 collected from two hospitals in Sri Lanka, UHKDU, and CNTH, to better understand the evolution of these viruses and how they relate to other DENV-3 strains globally. Within the relatively small sampling of our current cohorts, we have found two types of DENV-3 strains. Based on our analysis, there is a clear genetic divergence of one newly isolated virus from the other DENV-3 strains, suggesting that strains from 2017 and 2018 are the most predominant while one new strain likely originating from India is also circulating.

We conducted sequencing for DENV1 isolates for nine samples, seven strains are compatible with currently circulating strains of Sri Lanka in 2018 and two were found to be different. Further analysis showed these two closely related genotypes detected in China in 2014/2015 and in Sri Lanka in 2009/2010/2012. Phylogenetic trees were drawn using the strains with most identical bases for in-depth analysis of DENV-1, DENV-2 and DENV-3. Multiple nucleotide substitutions were observed with the DENV1 and DENV-3 sequences. Some mutations observed were comparable to other reported DENV1 strains (Fig 5), while some mutations are observed to be novel to sequences of this C-prM and E gene region. DENV 3 strains also showed the most mutations in the C-prM and E gene region (Fig 3).

Our analysis indicates less movement of people between countries and highlights the importance of better understanding the virus exchange dynamics between the neighbouring countries. We speculate this change in recent times may be due to the pandemic travel restrictions during the years 2020–2021 and as a result no dominant strains have entered the country and only the existing milder strains are co-circulating within the population. These sequence data will strengthen our ability to characterize the evolution and circulation patterns of the four dengue serotypes and it is important that these efforts continue. According to the literature, it was noted that in 2011, DENV-1 was dominant. In 2017 outbreak is due to DENV-2. According to our data, it shows that multiple strains are circulating during 2019 to 2022 and DENV-3 would have been dominant in 2023.

The S1 and S2 Figs show how the DENV-3 genotypes may have circulated among the nearby countries and the S3 Fig speculates as to how the DENV-2 genotype spread from Malaysia to other countries, from 2015 onwards and continues to circulate even in 2022. As shown in S6 Fig DENV1 has travelled from China to Italy in 2020 then eventually transferred to SL as well. Therefore, this data shows how new strains can enter countries to potentially cause new epidemics. It is important from a public health point of view to have ongoing surveillance in a country so that as soon as a new strain, not previously exposed to the population enters the country and starts to the authorities are notified and action can be taken to control the spread or prepare `for a large number of cases. Such as by having extra beds prepared in hospitals for a surge of dengue cases.

We find by sequencing, there were two genotypically distinct variants of DENV-3 isolated, one of which closely resembles strains isolated in India during 2016 and 2018 and the other one is an isolate which was present in Sri Lanka during 2017 onwards. We also found the same pattern for DENV-1 where two genotypically distinct variants of DENV-1 isolated, were found to closely resemble strains isolated in India during 2018 and other one is a currently circulating isolate which was present in Sri Lanka during 2009. No new variant was reported for the DENV-2 tree, suggesting that the old DENV-2 which caused the epidemic in 2017 is still circulating.

In summary, our data indicate that during the study period, Dengue virus (DENV) serotypes 1, 2, 3 and 4 were in circulation. We were only able to sequence DENV 1, DENV 2 and DENV 3 strains. These serotypes were found to have various genotypes circulating globally, particularly in Asian countries. Moreover, within the country under study, the same genotype was observed with minor variations. However, the emergence of new genotypes of DENV1 and DENV3 in a population that is relatively immune can potentially go unnoticed, leading to new outbreaks not being reported as previously experienced in the country. Consequently, it is crucial for vector control strategies to primarily target vector populations within the country. Additionally, early warning systems should be established in countries to monitor any changes in the circulating DENV serotypes.

It is important to monitor circulating serotypes to better understand dengue dynamics and prepare for potential outbreaks. The absence of a dengue outbreak during the COVID-19 pandemic may have been influenced by factors such as improved waste management practices and restricted travel, preventing the spread and introduction of new dominant strains, with the milder existing strains of the 4 serotypes expanding in the population. Considering that almost all the 4 serotypes have been circulating within the population, one would suspect immunity against all 4 serotypes has built up within the population, and we can speculate that this might be a reason for the absence of a large outbreak. Continued efforts in virologic surveillance and understanding serotype dynamics are essential for effective dengue control in Sri Lanka and globally.

## Supporting information

S1 Table Comparison of base pair similarity among sequences of DENV3. (TIFF)

S2 Table Comparison of base pair similarity among sequences of DENV1. (TIFF)

S1 Fig Predicted country to country movement of DENV3 circulation A. (Base map source: https://www.usgs.gov/maps/international-geomagnetic-reference-field-2005).
(TIF)

S2 Fig Predicted country to country movement of DENV3 circulation B. (Base map source: https://www.usgs.gov/maps/international-geomagnetic-reference-field-2005).
(TIF)

S3 Fig Predicted country to country movement of DENV2 circulation. (Base map source: https://www.usgs.gov/maps/international-geomagnetic-reference-field-2005).
(TIF)

S4 Fig Predicted country to country movement of DENV1 circulation A. (Base map source: https://www.usgs.gov/maps/international-geomagnetic-reference-field-2005).
(TIF)

S5 Fig Predicted country to country movement of DENV1 circulation B. (Base map source: https://www.usgs.gov/maps/international-geomagnetic-reference-field-2005).
(TIF)

S6 Fig Predicted country to country movement of DENV1 circulation C. (Base map source: https://www.usgs.gov/maps/international-geomagnetic-reference-field-2005).
(TIF)
