# Supplementary figures and images for: Identifying the strains of dengue circulating in the western province of Sri Lanka during 2019–2022
Source: PLOS Glob Public Health. 2024 Jul 16;4(7):e0003150. doi: 10.1371/journal.pgph.0003150 (PMC11251588; doi:10.1371/journal.pgph.0003150)

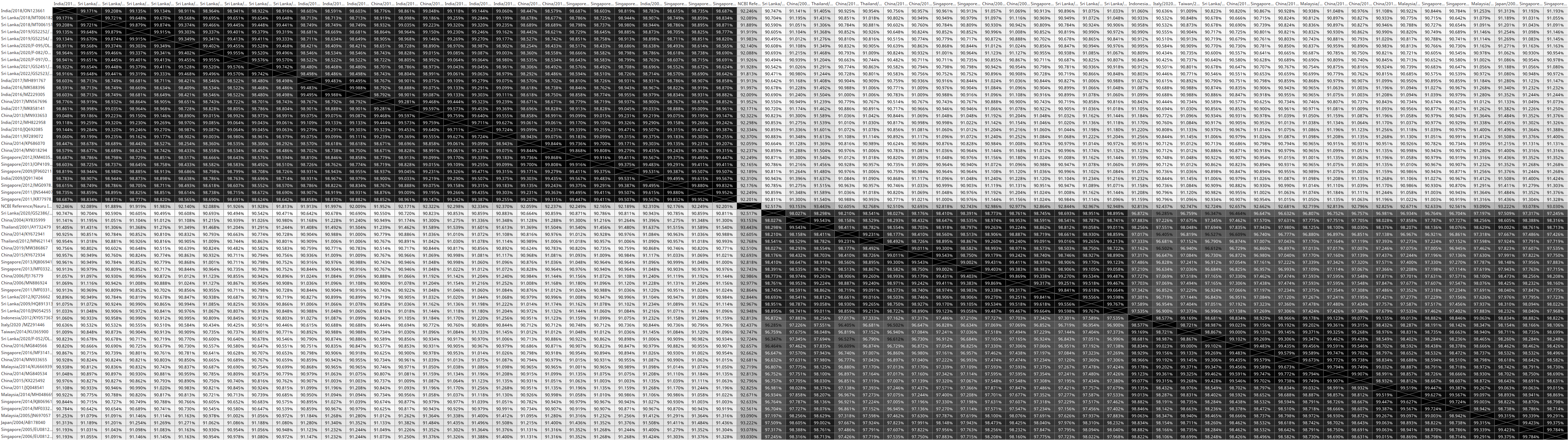

Supplement: S1 Table — (TIFF) [file pgph.0003150.s001.tiff]

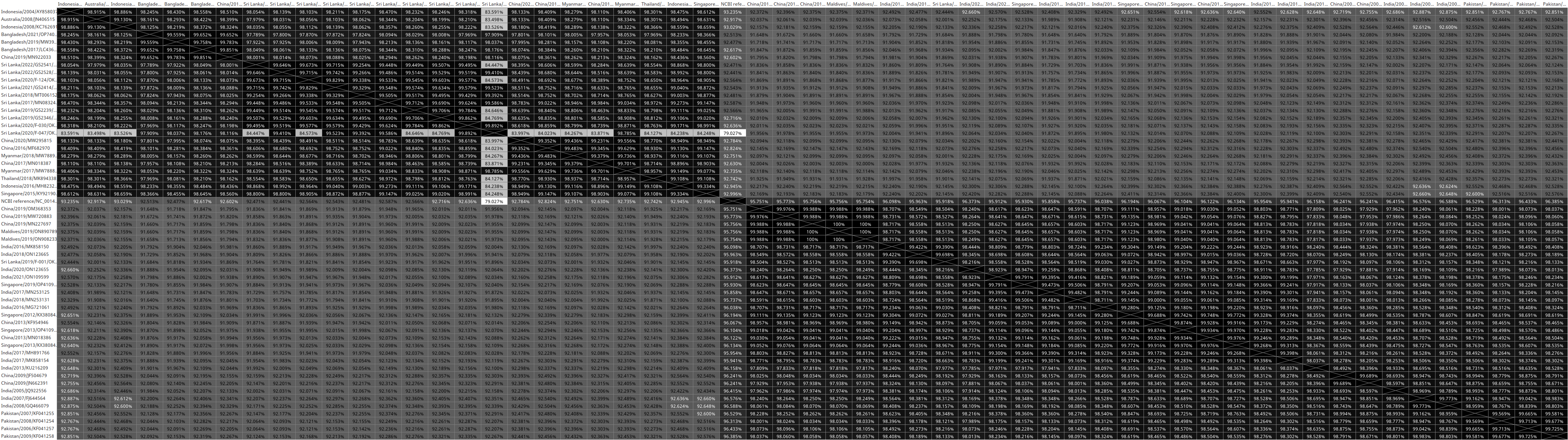

Supplement: S2 Table — (TIFF) [file pgph.0003150.s002.tiff]

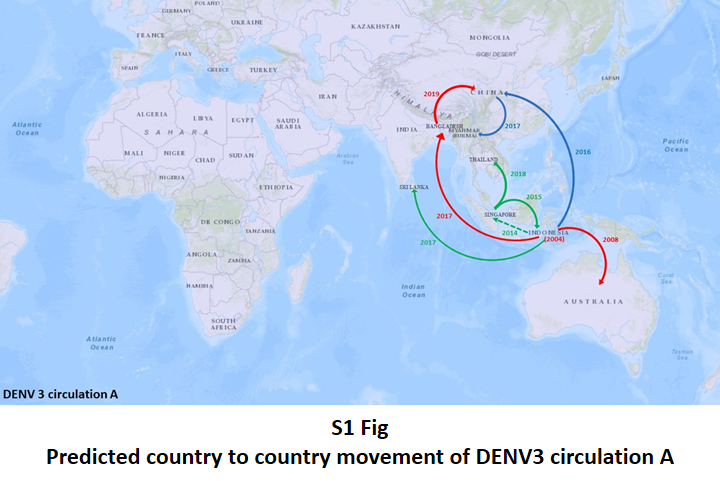

Supplement: S1 Fig — (Base map source: https://www.usgs.gov/maps/international-geomagnetic-reference-field-2005). (TIF) [file pgph.0003150.s003.TIF]

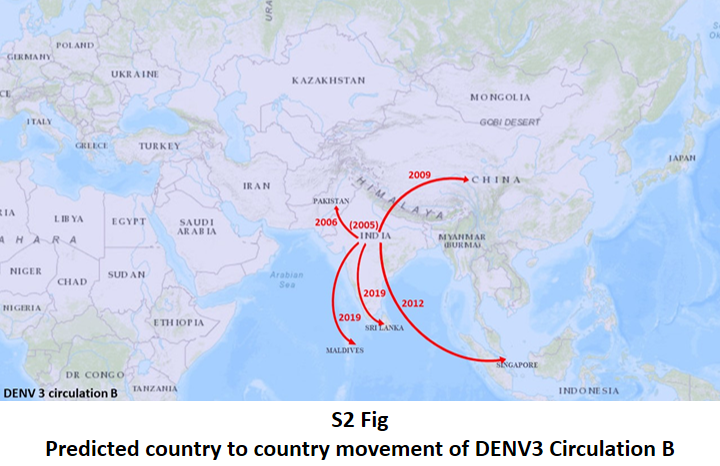

Supplement: S2 Fig — (Base map source: https://www.usgs.gov/maps/international-geomagnetic-reference-field-2005). (TIF) [file pgph.0003150.s004.TIF]

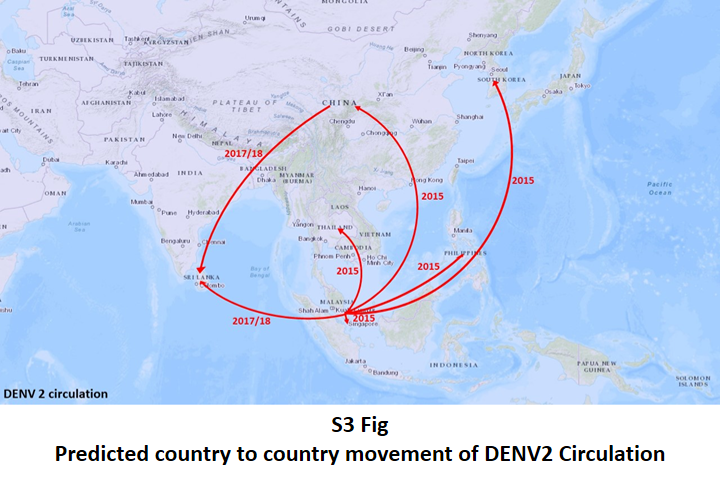

Supplement: S3 Fig — (Base map source: https://www.usgs.gov/maps/international-geomagnetic-reference-field-2005). (TIF) [file pgph.0003150.s005.TIF]

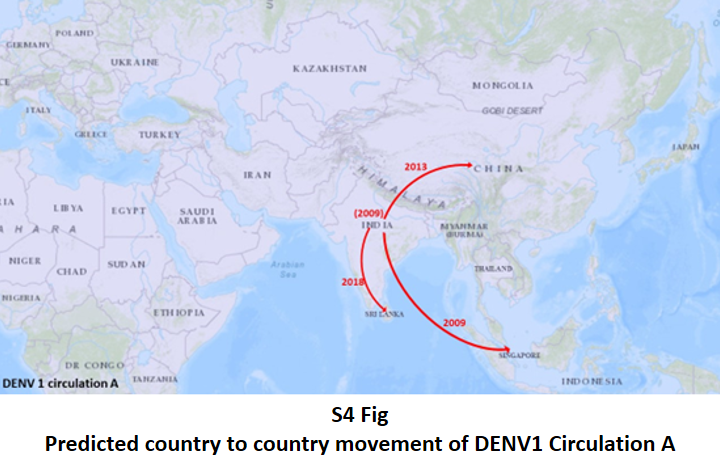

Supplement: S4 Fig — (Base map source: https://www.usgs.gov/maps/international-geomagnetic-reference-field-2005). (TIF) [file pgph.0003150.s006.TIF]

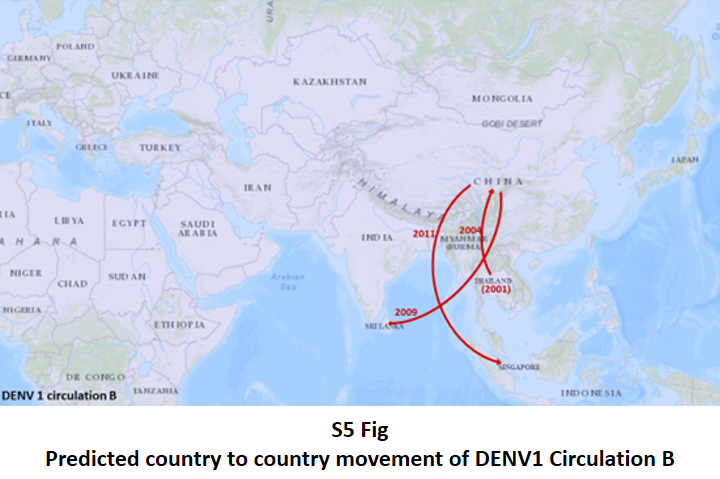

Supplement: S5 Fig — (Base map source: https://www.usgs.gov/maps/international-geomagnetic-reference-field-2005). (TIF) [file pgph.0003150.s007.TIF]

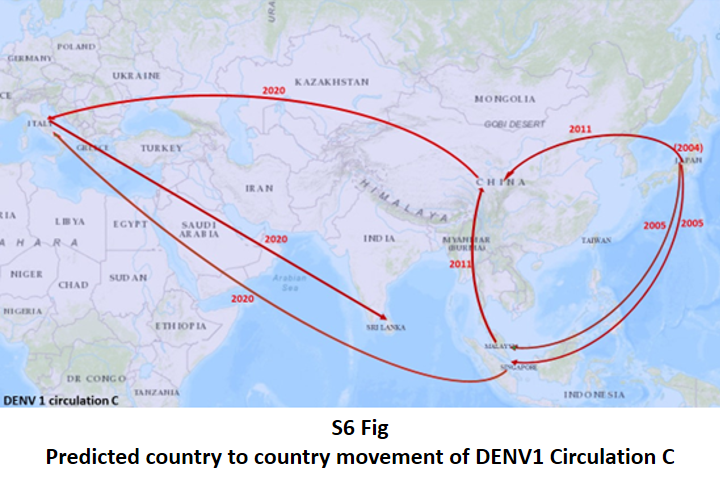

Supplement: S6 Fig — (Base map source: https://www.usgs.gov/maps/international-geomagnetic-reference-field-2005). (TIF) [file pgph.0003150.s008.TIF]
